# Population genomic analyses support sympatric origins of parapatric morphs in a salamander

**DOI:** 10.1002/ece3.9537

**Published:** 2022-11-27

**Authors:** Emily Buckingham, Jeffrey W. Streicher, M. Caitlin Fisher‐Reid, Tereza Jezkova, John J. Wiens

**Affiliations:** ^1^ Department of Life Sciences The Natural History Museum London UK; ^2^ Department of Life Sciences Imperial College London (South Kensington) London UK; ^3^ Department of Ecology and Evolutionary Biology University of Arizona Tucson Arizona USA; ^4^ Department of Biological Sciences Bridgewater State University Bridgewater Massachusetts USA; ^5^ Department of Biology Miami University Oxford Ohio USA

**Keywords:** amphibians, parapatry, salamanders, speciation, sympatry

## Abstract

In numerous clades, divergent sister species have largely non‐overlapping geographic ranges. This pattern presumably arises because species diverged in allopatry or parapatry, prior to a subsequent contact. Here, we provide population‐genomic evidence for the opposite scenario: previously sympatric ecotypes that have spatially separated into divergent monomorphic populations over large geographic scales (reverse sympatric scenario). We analyzed a North American salamander (*Plethodon cinereus*) with two color morphs that are broadly sympatric: striped (redback) and unstriped (leadback). Sympatric morphs can show considerable divergence in other traits, and many *Plethodon* species are fixed for a single morph. Long Island (New York) is unusual in having many pure redback and leadback populations that are spatially separated, with pure redback populations in the west and pure leadbacks in the east. Previous work showed that these pure‐morph populations were genetically, morphologically, and ecologically divergent. Here, we performed a coalescent‐based analysis of new data from 88,696 single‐nucleotide polymorphisms to address the origins of these populations. This analysis strongly supports the monophyly of Long Island populations and their subsequent divergence into pure redback and pure leadback populations. Taken together, these results suggest that the formerly sympatric mainland morphs separated into parapatric populations on Long Island, reversing the conventional speciation scenario.

## Graphical abstract

Under classic speciation models, species with divergent morphologies and ecologies are thought to arise either in situ (sympatric speciation), in geographic isolation (allopatric speciation), or in distinct but geographically adjacent habitats (parapatric speciation). In this study we provide evidence for a different situation that has rarely been discussed in the literature; the “reverse sympatric” scenario. We use a novel population‐genomic dataset from salamanders (*Plethodon cinereus*) on Long Island to provide evidence for this model.

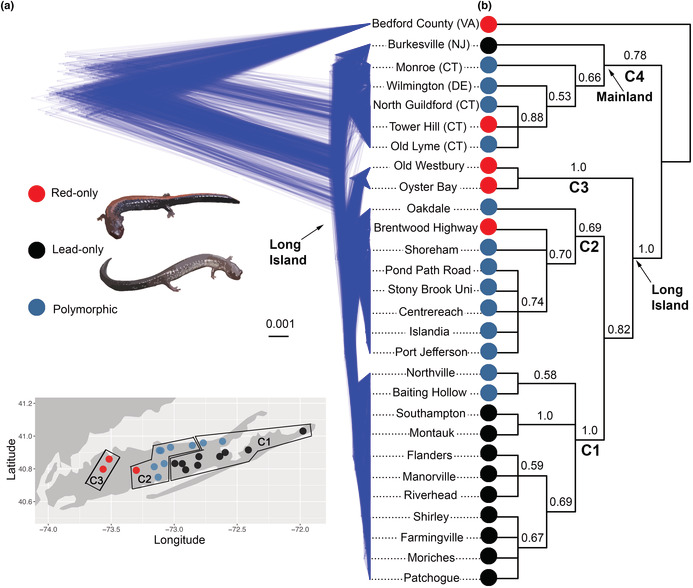

## 1 INTRODUCTION

**FIGURE 1:**
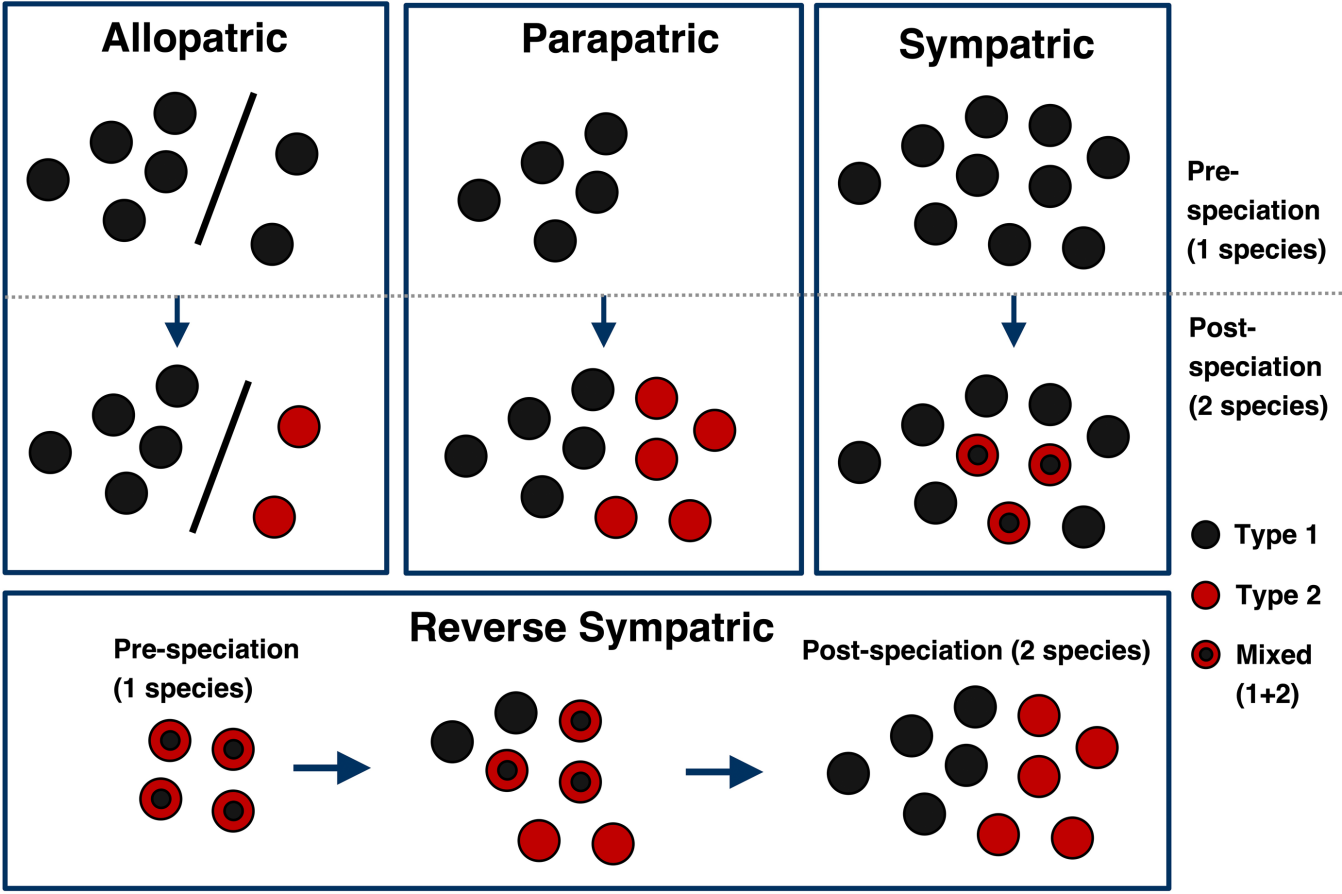
Hypothetical examples illustrating different geographic modes of speciation over time. The three traditionally recognized modes are shown on top. The bottom row shows the reverse‐sympatric scenario discussed here. Each circle represents a population. All the three modes also include divergence in some trait over time, starting with all populations fixed for type 1. Under sympatric speciation, populations eventually have both types present in sympatry (mixed populations: black circle with red ring). Under the reverse‐sympatric model, populations begin with both the types present in sympatry (mixed), but these two types become geographically sorted over time into two parapatrically distributed species.

A long‐standing question in evolutionary biology (Bolnick & Fitzpatrick, 2007; Coyne & Orr, 2004; Dobzhansky, 1937; Futuyma, 2013; Mayr, 1942, 1947; Skeels & Cardillo, 2019; Smith, 1966; White, 1978) is how new species arise geographically (Figure 1). Many species are thought to arise through the geographic separation of populations of a previously contiguous ancestral species (i.e., allopatric speciation; Coyne & Orr, 2004; Hernández‐Hernández et al., 2021). Others may arise through ecological divergence among adjacent populations (parapatric). Some species may arise within the range of another species (sympatric), but this mode is controversial and is considered rare (Coyne & Orr, 2004) or at least relatively uncommon (Hernández‐Hernández et al., 2021). Thus, many sister species with presently overlapping ranges are thought to have acquired this distribution through dispersal after speciation.

An intriguing possibility is that species that are currently allopatric or parapatric at large scales arose from spatial separation of sympatric ecotypes along an ecological gradient (hereafter “reverse sympatric speciation”; Figure 1). In some ways, this scenario turns the classic model of allopatric or parapatric divergence and secondary sympatry on its head. Specifically, under allopatric and parapatric speciation, divergence is thought to occur primarily in allopatry and parapatry (respectively), and not in sympatry. Under typical models of sympatric speciation, divergence, and speciation occur in sympatry, but post‐speciation the species remain in sympatry, rather than becoming parapatrically distributed, as in reverse‐sympatric speciation (Figure 1). Scenarios similar to reverse‐sympatric speciation have been suggested in some theoretical models (e.g., Doebeli & Dieckmann, 2000, 2003) and in some empirical examples at small spatial scales (e.g., Ingram, 2011; Seehausen et al., 2008). However, to our knowledge, this pattern has not been shown at the larger spatial scales typical of species' geographic ranges.

**FIGURE 2:**
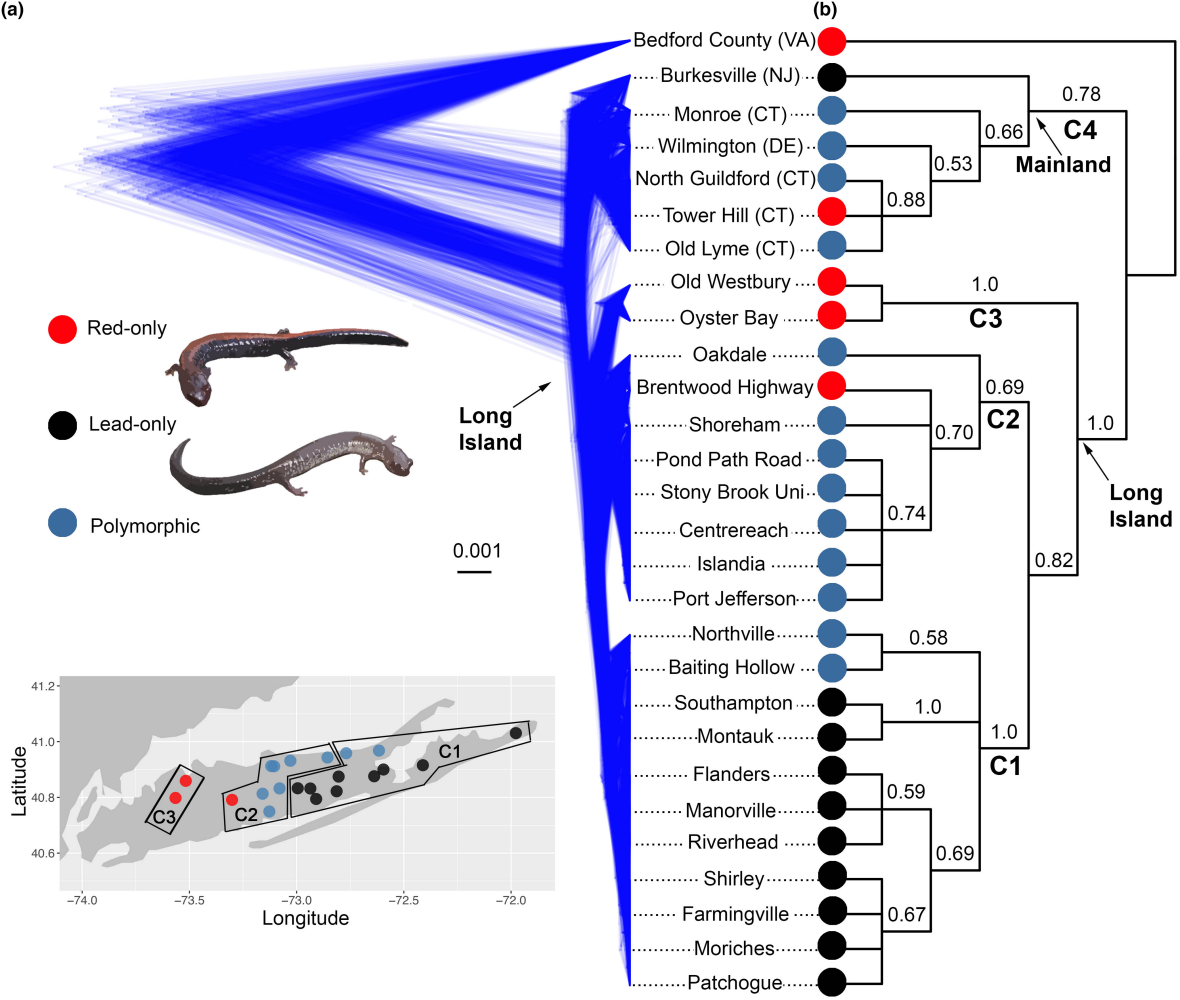
Population‐level phylogeny of *Plethodon cinereus* from Long Island (LI) and adjacent regions. Trees were inferred using the method SNAPP from 88,696 SNPs identified from ddRADseq data. An alternative tree based on 48,228 SNPs is shown in Figure S1. (a) Densitree overview with branch lengths equivalent to expected mutations and (b) majority‐rule consensus tree with posterior probabilities (with arbitrary branch lengths). Overlaid blue lines in the Densitree plot represent individual species trees (from the posterior distribution of the SNAPP analysis) and these all agree on the monophyly of LI populations and the monophyly of LI subclades C1 (mostly pure leadback populations) and C3 (pure redback populations). This support is also depicted in the majority‐rule consensus where these clades all have posterior probability of 1.0. However, there is extensive disagreement between trees over relationships among populations within these clades. Inset map depicts the sampling of LI populations for the present study (mainland localities not shown). Red dots indicate pure redback populations, black indicate pure leadback populations, and teal indicate populations with both morphs. Examples of leadback and redback morphs are also shown. Labeled clades (C1–C4) are discussed further in the text. Locality data are in Table S1.

One potential large‐scale example of this scenario (or important aspects of it) involves a salamander species (*Plethodon cinereus*) on Long Island (New York). This species is broadly distributed in eastern North America (Radomski et al., 2020) and has two common color morphs across its range (Cosentino et al., 2017): one with a red dorsal stripe (redback) and an unstriped morph (leadback; Figure 2). Morph frequencies vary across the geographic range, with mixed populations widespread along the mid‐Atlantic coast and higher frequencies of the redback morph at higher elevations and latitudes (Cosentino et al., 2017). These morphs show some ecological and behavioral differentiation within sympatric populations, including differences in thermal activity patterns (Anthony et al., 2008; Lotter & Scott, 1977; Moreno, 1989; but see Petruzzi et al., 2006) and some assortative mating (Anthony et al., 2008). On Long Island (LI hereafter), however, many populations are either pure leadback or pure redback (Figure 2). Moreover, these pure‐morph populations are geographically separated, with pure leadback populations in the eastern part of the island, pure redback in the west, and polymorphic populations in between (Fisher‐Reid et al., 2013; Williams et al., 1968). A previous study (Fisher‐Reid et al., 2013) found that these pure leadback and redback populations on LI are divergent ecologically (in microclimate and macroclimate), morphologically (in costal groove number, possibly related to burrowing behavior), and genetically (in microsatellites and an amino acid change in mitochondrial ATPase). However, it should also be emphasized that these two sets of populations do not appear to be distinct species at present (and might never be). Intriguingly, many other *Plethodon* species also show this redback/leadback polymorphism, whereas other species are fixed for the leadback or redback morph (Fisher‐Reid & Wiens, 2015). There are also interesting parallels between the ecological and morphological differences between color morphs within species and those among species (Fisher‐Reid & Wiens, 2015). Thus, within‐species divergence between morphs may culminate in divergence between species (although the colors themselves may not be directly involved; Fisher‐Reid et al., 2013; Fisher‐Reid & Wiens, 2015).

However, a weakness of the previous study on LI salamanders was that strong support was lacking for monophyly of LI populations (Fisher‐Reid et al., 2013). Monophyly of LI populations is crucial, because only this pattern is consistent with a single colonization of LI by a polymorphic population (which then segregated spatially into pure leadback and redback populations). Other phylogenetic patterns are potentially consistent with other scenarios. For example, repeated colonizations of LI by populations fixed for different morphs would not support the reverse‐sympatric scenario. The previous study found only weak support for LI monophyly (bootstrap <50%), presumably because few microsatellite loci were sampled (*n* = 7).

Here, we utilize population‐genomic analyses to test the monophyly of LI populations and the scenario of parapatric separation of sympatric ecotypes. We find strong support for monophyly of LI populations, and the subsequent divergence of pure leadback and redback populations after colonization (although not full speciation).

## 2 METHODS

### 2.1 Sampling design and data collection

Our primary sampling used 60 of the same individuals from the previous study of LI populations (Fisher‐Reid et al., 2013). DNA was extracted using either a Qiagen DNeasy kit or magnetic beads (Rohland & Reich, 2012). Data were collected using the double‐digest RADseq approach (Peterson et al., 2012; Streicher et al., 2014). High‐weight DNA was digested using two restriction enzymes, SbFI–HF and MspI (New England Biosciences; NEB). Enzymatic digestion was performed in 50 μl reactions (5 μl Cutsmart buffer, 1 μl each enzyme, 43 μl water) at 37°C for 8 h. Digestions were cleaned with 90 μl magnetic beads and eluted into 30 μl of TRIS. Custom adapters (Streicher et al., 2014) were ligated to digested fragments using T4 DNA Ligase (NEB) including 30 μl of cleaned digestions, 2 μl of P1 adapter, 2 μl P2 adapter, 4 μl ligation buffer (NEB), 1 μl ligase, and 1 μl water. Ligations were then pooled into sets of 10 samples (total volume of 400 μl), cleaned using 700 μl of magnetic bead solution, and size selected between 435–535 base pairs using a Pippin Prep (Sage Sciences). We used nested barcoding (indexing) to further combine sets of 10 samples into the final sequencing library. Polymerase chain reaction (using barcoded PCR primers; Streicher et al., 2014) was used to amplify size selected samples for 10 cycles. Sequencing was conducted at the University of Texas Southwestern Medical Center and the University of Arizona using Illumina® HiSeq platforms.

After removing four individuals with insufficient data (<10,000 sites genotyped), the primary dataset included 56 individuals, including 42 from LI (21 populations) and 14 (seven populations) from the mainland. LI sampling included three pure redback populations, nine polymorphic populations, and nine pure leadback (Figure 2). Note that sampling in far western LI is absent because of the heavy urbanization associated with New York City. We found salamanders only in natural habitat.

Sequence data were demultiplexed by PCR index using Illumina software and then the *process_radtags* script from STACKS 2.41 (Catchen et al., 2013). We also used *process_radtags* to remove low‐quality reads (‐q flag; if the average score of a read was below 90%, it was discarded). To identify SNPs (single‐nucleotide polymorphisms), we ran the “Core” pipeline (i.e. ustacks [‐m 3], cstacks [‐n 1], sstacks, tsv2bam, gstacks and populations), treating each individual as a population. We only used SNPs from the first paired‐end read to minimize linkage effects (Streicher et al., 2014).

### 2.2 Phylogenetic inference

We inferred population‐level phylogenies based on the coalescent model in SNAPP (Bryant et al., 2012) using the primary dataset. We first converted “structure” formatted files from STACKS 2.41 (Catchen et al., 2013) into a binary nexus format using PGDSpider (Lischer & Excoffier, 2012). SNAPP does not allow missing data but does allow for multiple individuals to be used as terminal taxa. Increasing the number of individuals per tip decreases missing data across tips. Therefore, we only included populations with two or more individuals sampled (28 populations total). The sampled populations span the overall distribution of populations and color morphs on LI. We used the SNAPP template within the BEAUTi program in BEAST 2.6.3 (Bouckaert et al., 2019) to convert the nexus file to an XML input file. The XML file was then run in BEAST.

SNAPP 2.4.1 analyses assume two qualities of SNPs: (i) each polymorphism is a biallelic character and (ii) that their genealogies have very little linkage (Bryant et al., 2012). Our dataset satisfies the first requirement because it is comprised exclusively of biallelic SNPs. Using SNPs from only the first paired‐end read should substantially reduce linkage among SNPs because it excludes all SNPs on the second paired‐end read from each RAD locus. Nevertheless we further explored the effect of linkage by running two separate phylogenetic analyses: (i) using only the first SNP from each RAD locus (hereafter first SNP‐only) to reduce linkage, and (ii) using all SNPs from the first paired‐end read (hereafter all SNPs) which increases the size of the data matrix but also the amount of linkage among SNPs. In both SNAPP analyses, we used 4 million generations, sampling every 1000 generations. We removed 60% of the posterior distribution of samples as burnin. This was the point at which effective sample sizes (ESS) were >100 for key statistics (i.e., posterior, *u*, and *v* statistics). We determined ESS using Tracer v1.7.1 (from https://github.com/beast‐dev/tracer/releases).

We visualized results using the R packages phangorn 2.2 (Schliep, 2011) and ape 5.0 (Paradis & Schliep, 2019). To summarize the posterior distribution of trees from SNAPP we used the “densiTree” function of phangorn and a majority‐rule consensus tree was constructed using the “consensus” function of ape. We used DensiTree 2.2.7 (Bouckaert & Heled, 2014) to obtain posterior probabilities of clades (PPs), which are the standard measure of branch support for SNAPP.

**FIGURE 3:**
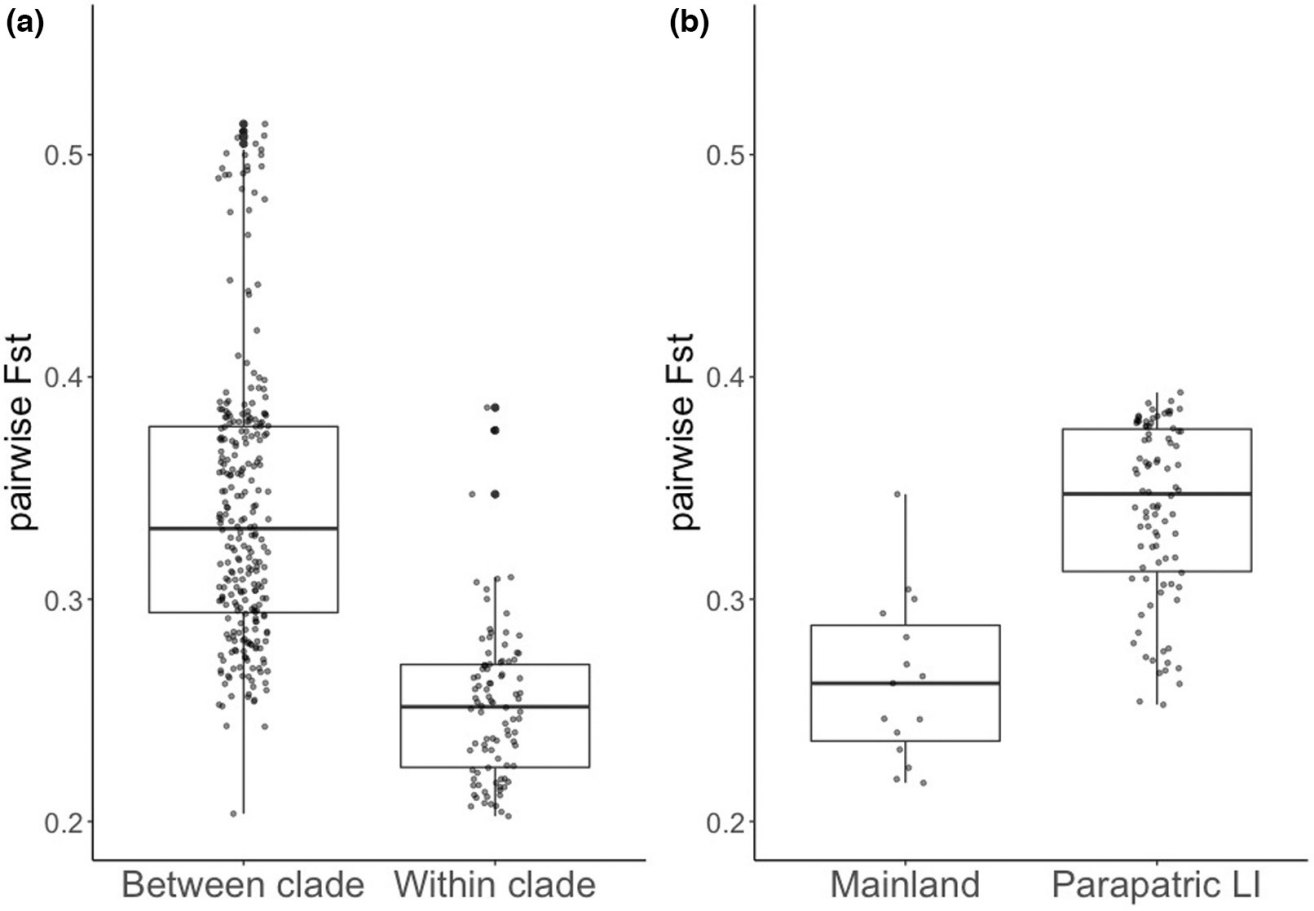
Results of *F*_ST_ pairwise comparisons. (a) Between‐clade versus within‐clade pairwise *F*_ST_ scores for clades C1–C4 from Figure 2. (b) Between‐mainland populations (clade C4) versus between‐parapatric Long Island (LI) clades. Between‐paraptric LI clade scores are from comparisons of clade C2 (polymorphic) to clade C3 (pure redback) and clade C2 to clade C1 (mostly leadback). The Kruskall–Wallis test results are in Table S3.

The SNAPP analysis also assumes that incongruence among loci is explained by incomplete lineage sorting and that no gene flow is occurring (Bryant et al., 2012). Gene flow almost certainly occurred among some sampled *P. cinereus* populations, but this is also true in many previous studies where SNAPP was used (e.g., Foote & Morin, 2016; Prates et al., 2018; Streicher et al., 2014). The effect of gene flow on phylogenetic inference from SNAPP is that it shortens branch lengths, reduces node support, and results in populations being grouped together based on the extent of gene flow among them (Foote & Morin, 2016; Leaché et al., 2014). Our inferred phylogenies (Figure 2; Figure S1) contained multiple clades with maximal PP (=1.0). Furthermore, fixation indices (*F*_ST_; Wright, 1951) suggested less genetic connectivity among these clades than within them (see Section 3, Figure 3a). Therefore, it appears that gene flow did not erase phylogeographic patterns among the sampled populations.

### 2.3 Genetic divergence and heterozygosity

We estimated genetic divergence among populations and morphs via pairwise *F*_ST_ scores generated using STACKS 2.41 (Catchen et al., 2013). We compared within‐clade versus between‐clade *F*_ST_ scores based on the tree (clades C1–C4; Figure 2). We predicted lower *F*_ST_ scores within clades than among clades, assuming these clades show reduced gene flow, and that this reduced gene flow is related to genetic divergence among populations. We also compared pairwise *F*_ST_ scores between (i) continuous mainland populations (within clade C4) and (ii) between parapatric clades on LI (pure redback [C3] vs. polymorphic [C2] and mostly leadback [C1] vs. polymorphic [C2]). We predicted that *F*_ST_ scores between adjacent but divergent LI clades (pure vs. polymorphic) would be higher than *F*_ST_ scores between geographically distant mainland populations of the same type (polymorphic). Thus, we expected more genetic divergence between these divergent parapatric LI populations (<50 km apart) than between continuously distributed mainland populations separated by ~340 km. We used non‐parametric Kruskall–Wallis tests, implemented in R version 3.5.1 (R Core Team, 2018) to test all *F*_ST_ predictions.

To place pairwise *F*_ST_ scores in a direct geographic context, we plotted them against pairwise geographic distances (in km) to test for two patterns that would support the occurrence of incipient speciation on LI. First, we expected that populations on LI separated by similar geographic distances would have higher *F*_ST_ scores when they are from different clades (between‐clade comparisons) compared to when they belong to the same clade (within‐clade comparisons). Second, we expected that mainland populations separated by distances greater than the size of LI would have lower *F*_ST_ scores than parapatrically distributed LI clades.

For these analyses, we included only mainland populations from the northeastern US (NE mainland). These populations are geographically adjacent to LI and belong to the same clade of closely related populations as LI populations do (Radomski et al., 2020). We did not include the population from Bedford County, Virginia in these comparisons because it is only distantly related to both the NE mainland and LI populations (Radomski et al., 2020).

We used the geodist R package (Karney, 2013; Padgham & Sumner, 2021) to calculate pairwise geographic distances. To assess the strength of correlation between pairwise *F*_ST_ scores and pairwise geographic distances we performed Mantel tests on three sets of distance matrices: (i) LI only, (ii) LI + NE mainland (clade C4; Figure 2), and (iii) NE mainland‐only. Mantel tests were conducted in the vegan R package (Oksanen et al., 2020) using Pearson's product–moment correlation and 999 permutations.

We also used non‐parametric Wilcoxon rank‐sum tests in R 3.5.1 to test for differences in heterozygosity and private alleles among sampled populations. Prior to estimating population genetic statistics, we further quality filtered the results from STACKS 2.41 (Catchen et al., 2013) and only included individuals with >500,000 sites genotyped. We arbitrarily selected this number of sites to ensure that estimates of genetic diversity were made from comparisons of individuals with large and similar amounts of data. We then used observed heterozygosity and private allele estimates from the “populations.sumstats_summary” file to conduct various tests. We predicted that populations on LI experienced a genetic bottleneck following their isolation on LI, which should have resulted in decreased population size, leading to individuals with lower observed heterozygosity and fewer private alleles (compared to mainland populations).

We were able to include 13 additional samples from Fisher‐Reid et al. (2013) for individual private allele and heterozygosity estimates (Table S1). This was enabled by leveraging another RADseq dataset of *P. cinereus* that was generated using a different size‐selection protocol (~335–435 base pairs). We examined our datasets for batch effects that might mislead our interpretations and found that our results were robust (Appendix S1).

### 2.4 Reconstructing range expansion

We used the neutral expectations of genetic drift to test for a possible signature of range expansion on LI. Given that western LI is closer to the mainland than eastern LI (Figure 2), we predicted that range expansion occurred from west to east. We used a Kruskal–Wallis test to determine if pure redback populations in western LI had significantly more private alleles than their polymorphic and pure leadback counterparts, as predicted given eastward expansion. We also used the R package rangeExpansion (Peter & Slatkin, 2013) to calculate pairwise directionality indices (ψ; Peter & Slatkin, 2013; Streicher et al., 2016). We generally used the same sampling as the SNAPP analyses of the primary dataset. However, we added two LI pure redback populations (Selden and Woodbury; Table S1) that were excluded from the phylogenetic analyses because they were only represented by a single individual. Two populations of *P. cinereus* from Virginia were used as outgroups when calculating ψ indices.

Range expansion leads to a series of founder effects that allow both deleterious and recessive alleles to be fixed within a population, resulting in newly founded populations being genetically different from the source population (Peter & Slatkin, 2013). The consequences of this process include reduced numbers of private alleles and lower heterozygosity in populations on the expansion front compared with those closest to the ancestral population (the origin of expansion). Compared with observed heterozygosity (which was not significantly different across LI populations; Table 1), the ψ index can be a more sensitive test for detecting the geographic origins of a range expansion. The pairwise ψ score is generated from comparison of two populations, labeled *S*_1_ and *S*_2_. Positive ψ values indicate stronger founder effects on *S*_2_ and thus a greater geographical distance from the source population, whereas negative values indicate closer proximity to the origin of expansion in *S*_2_ (Streicher et al., 2016).

**TABLE 1:** Summary statistics and results for four population‐level clades (Figure 2)

Clade	Individuals	Populations	Average heterozygosity (SD)	Average private alleles (SD)	Average within‐group *F*_ST_ (SD)	Average number of sites (SD)
C1. LI (mostly leadback)	22	11	0.0004 (<0.0001)	7.2 (3.3)	0.231 (0.04)	1,439,745 (69,720)
C2. LI (polymorphic)	16	8	0.0003 (<0.0001)	9.6 (6.1)	0.221 (0.03)	1,402,575 (134,491)
C3. LI (pure redback)	4	2	0.0003 (0.0001)	73.3 (110.4)	0.244 (0.01)	1,040,084 (203,029)
C4. NE Mainland	12	6	0.0006 (0.0001)	62.8 (112.6)	0.280 (0.05)	1,134,125 (401,152)

*Note*: See Table S1 for additional information. Average number of sites is the number of nucleotides identified as belonging to RAD loci using the STACKS 2.41 pipeline.

Given our prediction that range expansion on LI should have occurred in a mostly west‐to‐east direction, we had clear expectations for the patterns in pairwise ψ scores. Specifically, more easterly populations in the *S*_2_ position should result in positive pairwise ψ scores. For example, pure leadback populations should, on average, have positive pairwise ψ scores when they are in the *S*_2_ position and pure redback and polymorphic populations are in the *S*_1_ position whereas pairwise ψ scores should be negative when pure redback and polymorphic populations are in the *S*_2_ position and pure leadback populations are in the *S*_1_ position. We compared different categories of pairwise ψ scores for the six possible combinations of pure leadback, pure redback, and polymorphic populations occupying the *S*_1_ and *S*_2_ positions. We used an ANOVA with Tukey posthoc pairwise comparisons to test for significant differences between mean pairwise ψ scores of these categories, and then assessed if statistically significant differences met our expectations of west‐to‐east range expansion.

We also investigated the robustness of these range expansion inferences to missing data. We found that different thresholds for including missing data all produced similar results (Appendix S1).

## 3 RESULTS

### 3.1 Phylogenetic analyses support the monophyly of Long Island populations

The SNAPP analyses were based on 48,228 unique RAD loci which resulted in data matrices of (i) 48,228 SNPs (first SNP‐only; 66.81% missing cells overall) and (ii) 88,696 SNPs (all SNPs; 52.06% missing cells). Both analyses supported the monophyly of sampled LI populations, one strongly (all SNPs; PP = 1.0; Figure 2) and one moderately (first SNP‐only: PP = 0.70; Figure S1). Both analyses also supported four clades on LI (PPs presented as: all SNPs: SNP analysis/first SNP‐only analysis): (i) an eastern LI clade containing mostly pure lead populations (C1, PP = 1.0/1.0), (ii) a central LI clade containing mostly polymorphic populations (C2, PP = 0.69/1.0), (iii) a western LI clade containing two pure redback populations (C3, PP = 1.0/1.0), and (iv) a clade of mostly polymorphic NE mainland populations (C4, PP = 0.78/0.83). Both consensus trees were consistent with eastward range expansion on LI, with the clade of pure redback populations from western LI placed as sister to other LI populations (Figure 2; Figure S1).

Interestingly, we found that the smaller data matrix with ostensibly less linkage had more well‐supported clades than the larger dataset (11 clades vs. 8 clades with PP > 0.70; Figure 2; Figure S1). Thus, the analysis with 88,696 SNPs had strong support for the monophyly of LI populations, but fewer well‐supported clades overall.

### 3.2 Estimates of genetic divergence

We sampled an average of 1.22 million sites per individual (± 374,478 SD), and found estimates of genetic diversity that were consistent with the phylogenetic patterns. Furthermore, *F*_ST_ scores were lower within clades than between clades (*H* = 171.9, *p* < .0001; Figure 3; Table S3). As predicted given incipient speciation between LI morphs, pairwise *F*_ST_ scores between parapatric LI clades (mostly leadback C1 vs. polymorphic C2 and pure redback C3 vs. polymorphic C2; Figure 2) were significantly higher than between mainland populations (C4, *H* = 26.2, *p* < .0001; Figure 3; Table S3). This occurred despite much greater maximum distances between mainland populations. For example, *F*_ST_ was 0.22 between populations from Wilmington, Delaware and Tower Hill, Connecticut (separated by ~320 km) whereas average pairwise *F*_ST_ among parapatric LI populations was 0.34 (±0.04 SD, separated by a maximum distance of ~100 km [Montauk to Centereach]).

**FIGURE 4:**
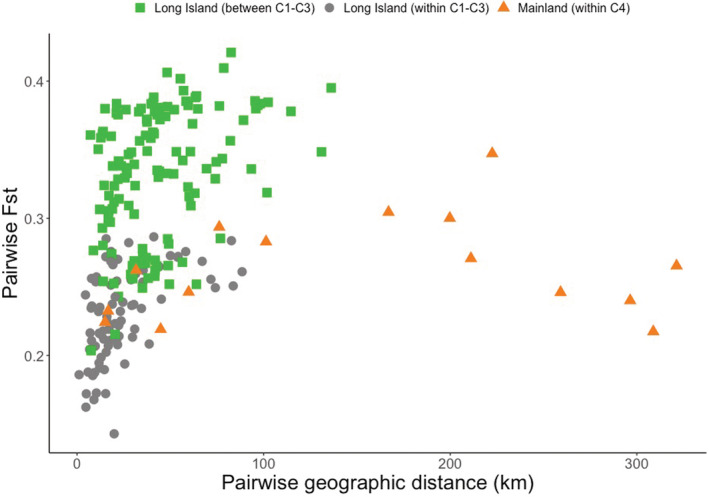
Results of *F*_ST_ pairwise comparisons in relation to geographic distance among LI and adjacent mainland populations. Comparisons among LI populations are indicated as within (circles) and between (squares) clades (C1–C3; Figure 2). Mainland comparisons indicated (triangles) are those within clade C4 (Figure 2).

Comparisons of pairwise *F*_ST_ scores and geographic distances further validated the phylogenetic patterns and stand‐alone *F*_ST_ score analyses (Figure 4). Specifically, *F*_ST_ scores were notably higher in the between‐LI‐clade comparisons than the within‐LI‐clade comparisons, for populations separated by similar geographic distances. We also observed that adjacent mainland populations had lower *F*_ST_ scores at greater distances relative to the comparisons between LI clades (Figure 4). Mantel tests revealed significant correlations between geographic distance and genetic divergence in the LI‐only dataset (Table 2). There was a weaker correlation in the analysis that combined LI‐only and NE mainland populations (Table 2). There was no significant relationship between pairwise *F*_ST_ scores and pairwise geographic distances in the NE mainland‐only dataset. However, our sample sizes were small for these latter comparisons (*n* = 36 individuals, Table 2).

**TABLE 2:** Mantel test results for correlations between pairwise *F*_ST_ scores and pairwise geographic distances (in km) for three datasets based on populations of *Plethodon cinereus* originating from Long Island (LI) and the northeastern (NE) mainland.

Dataset	Number of comparisons (cells in matrix)	Mantel *R* statistic	*p*
LI‐only	441	0.52	.001
LI + NE Mainland	729	0.26	.031
NE Mainland only	36	0.32	.317

### 3.3 Estimates of heterozygosity and range expansion

**FIGURE 5:**
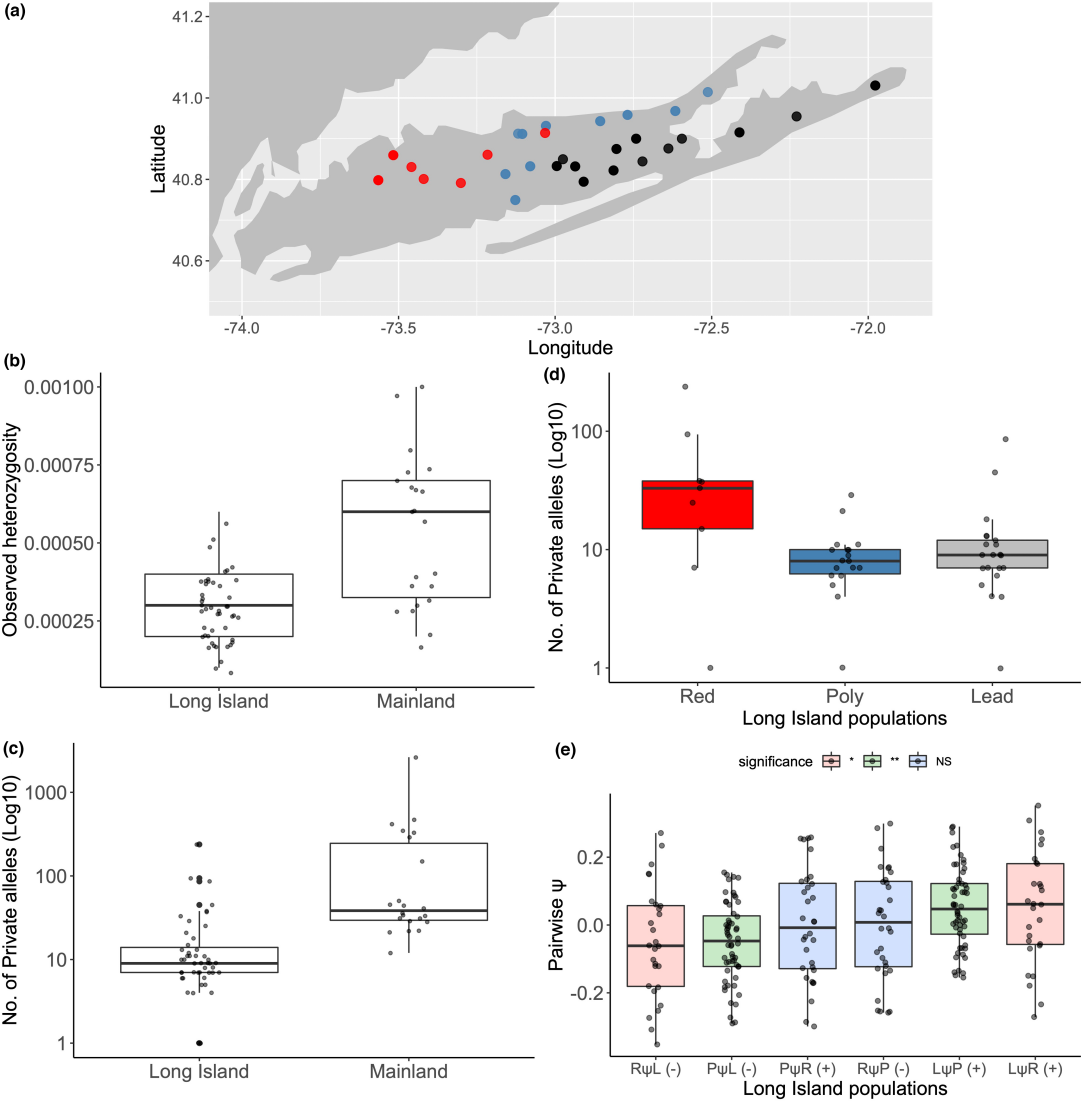
Results of analyses of heterozygosity, private alleles, and range expansion indices. (a) Distribution of *Plethodon cinereus* populations sampled across LI for heterozygosity and private‐allele analyses. Red dots indicate pure redback populations, black dots indicate pure leadback, and teal dots indicate polymorphic populations where both morphs occur. Sampling for range‐expansion analyses is shown in Figure 2. (b) Observed heterozygosity between individuals from Long Island (LI; *n* = 47) versus the mainland (*n* = 22). (c) Log_10_‐transformed number of private alleles in individuals from LI (*n* = 47) and the mainland (*n* = 22). (d) Number of private alleles among LI populations with different morphotype frequencies (Red = pure redback populations, *n* = 9; Poly = polymorphic populations, *n* = 18; Lead = pure leadback populations, *n* = 21). (e) Pairwise psi (ψ) statistics from the range‐expansion analyses. On the *x*‐axis, each category depicts comparisons where the population on the left of the ψ symbol was *S*_2_ and the population to the right of the symbol was *S*_1_; L = pure leadback populations; P = polymorphic populations; R = pure redback populations. Based on a hypothesis of range expansion from west to east, our expectations for whether pairwise mean ψ scores should be positive (+) or negative (−) is indicated in parentheses next to each *x*‐axis comparison category. Significant differences among pairwise mean ψ scores determined using the ANOVA and Tukey posthoc comparisons are indicated by boxplot colors (red = leadback‐redback comparisons; green = polymorphic‐leadback; blue = polymorphic‐redback) (*p = .026; **p = .003; NS, not significant).

Comparing NE mainland and LI samples (Figure 5b,c), individuals from LI had lower levels of heterozygosity (*W* = 197.5, *p* < .0001) and fewer private alleles (*W* = 113.5, *p* < .0001). This is consistent with a bottleneck in genetic diversity in the ancestral population on LI, and LI monophyly. Among LI populations, the number of private alleles was higher in pure redback populations than polymorphic or pure leadback populations (*H* = 11.2, *p* = .004; Table 1), consistent with range expansion occurring from west to east (Figure 5d).

Our analyses of ψ scores also supported our predictions for west‐to‐east expansion. As predicted, mean pairwise ψ scores for comparisons that had pure leadback populations in the *S*_2_ position and polymorphic or pure redback populations in the *S*_1_ position were significantly higher than the inverse comparisons (Figure 5e; TukeyHSD; PψL ~ LψP, *p* = .003 and RψL ~ LψR, *p* = .026). We did not observe significant differences in the other comparisons we made. All the range‐expansion reconstructions supported the origin of expansion being positioned substantially to the west of the geographical midpoint of our sampling on LI, consistent with our other results (Table S2).

## 4 DISCUSSION

The most well‐known geographic scenarios of speciation (Figure 1) suggest that populations generally diverge ecologically, morphologically, and genetically after becoming allopatric (allopatric speciation), or else that this divergence occurs as parapatric or sympatric populations speciate (Coyne & Orr, 2004). Here, we provide evidence for an unusual scenario in which initially sympatric ecotypes appear to have become parapatrically distributed and have diverged over relatively large spatial scales (Figure 2). Although these parapatric populations do not appear to be distinct species, they are ecologically, morphologically, and genetically divergent (Fisher‐Reid et al., 2013). This finding raises the possibility that some partially overlapping or parapatric species might have begun as sympatric ecotypes. We emphasize that this scenario is not new to our study: similar scenarios have been in the literature for >20 years (Doebeli & Dieckmann, 2000, 2003). However, previous empirical examples have been at small spatial scales (e.g., Ingram, 2011; Seehausen et al., 2008) and not at the relatively large scale analyzed here. Moreover, we recognize that some might consider the reverse‐sympatric scenario to be a type of parapatric speciation or sympatric speciation. We do not think that it fits well in either category, which is why we highlight it here as potentially distinct. Most importantly, under sympatric and parapatric speciation, species originate in sympatry and parapatry (respectively), whereas under reverse‐sympatric speciation, incipient species begin to differentiate in sympatry and then become parapatrically distributed (Figure 1).

This reverse‐sympatric scenario discussed here has other notable differences from the typical model of parapatric speciation along an ecological gradient (Coyne & Orr, 2004; Futuyma, 2013). The typical parapatric scenario starts with the ancestral population in the ancestral environment, which then colonizes a novel environment (Figure 1). The invasion of the novel environment might involve de novo mutations or standing genetic variation (e.g., Barrett & Schluter, 2008) or new combinations of existing variation (Marques et al., 2019). In the scenario here, the surprising part is not the invasion of a novel environment, but that populations at both ends of the gradient have diverged relative to the ancestral populations, leading to formerly sympatric ecotypes becoming parapatrically distributed. Here, the pine barrens of eastern LI may be the novel environment (Fisher‐Reid et al., 2013). Our new analyses here show that these pure leadback populations represent recent range expansion from western LI. These pure leadback populations have diverged relative to other LI and mainland populations in several ways, including in microclimate, macroclimate, morphology (costal groove number), and a nonsynonymous mitochondrial change (Fisher‐Reid et al., 2013). This is consistent with the typical parapatric speciation scenario. What is more surprising is that the western populations have also diverged, becoming pure redback (rather than polymorphic, as on the mainland). Moreover, these western populations also show higher genetic diversity (more private alleles; Figure 5d) than eastern populations, suggesting that their divergence is not simply explained by genetic drift in unusually small populations. There is no evidence that different LI populations were previously separated from each other by higher sea levels (Fisher‐Reid et al., 2013). Furthermore, the sympatric morphs on the mainland already differ in a suite of traits and show some assortative mating, at least in some populations (Acord et al., 2013; Anthony et al., 2008). These morphs also differ in microclimatic preferences, both on the mainland and on LI, and their parapatric distribution across LI is significantly related to macroclimatic patterns (Fisher‐Reid et al., 2013). However, the major genetic divergence between these morphs is among parapatric populations on LI, and not in sympatry on the mainland or LI. The reverse‐sympatric scenario is not simply spatial sorting of morphs that were already fully divergent. Thus, we are suggesting that there may be incipient speciation on LI among the now parapatric populations, but not necessarily among the sympatric morphs on the mainland (despite some assortative mating; Acord et al., 2013; Anthony et al., 2008) nor among sympatric morphs on LI.

This divergence on LI almost certainly occurred only within the last 50,000 years, given the age of northeastern U.S. populations in large‐scale phylogeographic analyses within *P. cinereus* (Radomski et al., 2020). Furthermore, based on geological evidence, LI was only formed in the last 25,000–30,000 years, and may have only been accessible to these salamanders in the last 10,000–15,000 years (Williams et al., 1968).

What might explain this unusual scenario on LI? Previous analyses showed that LI populations are macroclimatically distinct from adjacent mainland populations, such that both pure redback and pure leadback populations occur in novel climate space (Fisher‐Reid et al., 2013). Intriguingly, the spatial separation into pure redback and pure leadback populations has also occurred in other regions, including the coastal Delmarva peninsula (in Delaware‐Maryland‐Virginia; Highton, 1977). There, the separation of populations into pure leadback and pure redback populations shows similar correlations with macroclimate as those found on LI (Fisher‐Reid et al., 2013). However, different patterns may be present in other parts of the species' range (Evans et al., 2018), especially further west (Hantak et al., 2019). In summary, the fact that the scenario of segregation into pure redback and leadback populations is repeated under similar ecological conditions (i.e., coastal populations isolated from the mainland), with similar correlations between climate and morph frequencies, further suggests that the geographic separation into leadback and redback populations on LI is not simply random nor due to genetic drift.

The scenario of reverse‐sympatric speciation is also distinct from gene surfing, spatial sorting, and combinatorial speciation. It differs from genetic surfing (Edmonds et al., 2004; Hallatschek et al., 2007; Streicher et al., 2016) in that gene surfing involves a limited number of linked loci, whereas reverse‐sympatric speciation potentially involves phenotypic traits (and presumably many unlinked genes). For example, the LI redback and leadback populations have diverged in nuclear loci sampled throughout the genome, in a nonsynonymous mitochondrial marker, in macroclimate and microclimate, and in color and costal‐groove number (Fisher‐Reid et al., 2013). Reverse‐sympatric speciation differs from spatial sorting of phenotypes among populations (Shine et al., 2011) in that it is not necessarily tied to range‐expansion ability. Again, the pure leadback populations on eastern LI are divergent genetically, morphologically, and ecologically from pure redback LI populations and polymorphic mainland populations (Fisher‐Reid et al., 2013). This is inconsistent with the idea that these leadback populations are simply individuals from polymorphic populations that traveled east more rapidly (although leadback individuals do appear to disperse more than redbacks in a sympatric population in Maryland; Grant & Liebgold, 2017). Also, the pure leadback and redback populations occur under distinct ecological conditions, relative to each other and adjacent mainland populations (Fisher‐Reid et al., 2013). Finally, in *P. cinereus* on LI, there was divergence on both ends of their expansion across LI (i.e., pure redback populations in the west, pure leadback in the east), relative to the ancestral, polymorphic populations. Spatial sorting might help explain the pure leadback populations in eastern LI but fails to explain why the western populations are pure redback and not polymorphic. Furthermore, reverse‐sympatric speciation is not simply combinatorial speciation (Marques et al., 2019) since that hypothesis involves new combinations of existing genetic variation but is agnostic about geographic modes. Reverse‐sympatric speciation is clearly about geographic modes but is agnostic about the role of new combinations of existing genetic variation. Nevertheless, the presence of relevant, pre‐existing phenotypic variation is very important to this scenario (Figure 1).

We acknowledge that our sampling of mainland populations is not comprehensive. However, extensive range‐wide analyses of the phylogeography of *P. cinereus* (Radomski et al., 2020) suggest that LI populations belong to a clade of very closely related populations from the northeastern U.S. (Delaware and eastern Pennsylvania to Maine) and adjacent Canada. Thus, colonization of LI was almost certainly from adjacent areas in the northeast U.S., and not some other part of the species' range. Furthermore, our sampling of populations in adjacent Connecticut, New Jersey, and New York is limited (six populations sampled here). However, Fisher‐Reid et al. (2013) sampled these same six populations and seven additional ones from these three states (along with Delaware and Pennsylvania) and found results consistent with those here (i.e., monophyly of LI populations). Most importantly, it is unclear how sampling additional populations from the mainland could overturn our support for LI monophyly. For example, if one or a few unsampled populations from mainland Connecticut were found to be more closely related to some LI populations than to other mainland populations, this would suggest re‐colonization of the mainland, not multiple colonizations of LI. Conversely, if additional sampled mainland populations were more closely related to other mainland populations instead, this would have no impact on our conclusions.

We also note that we have not proven that the most recent common ancestor of LI populations was polymorphic (i.e., with both leadback and redback individuals). However, this was previously tested and supported by ancestral‐state reconstructions among LI and mainland populations (Fisher‐Reid et al., 2013). Indeed, most sampled populations adjacent to LI are polymorphic (Figure 2b; Fisher‐Reid et al., 2013), especially when morph frequencies are assessed based on large sample sizes (Lotter & Scott, 1977). The alternative explanation is that LI was colonized by a population consisting of only one morph (e.g., redback), and that the other morph then evolved independently on LI. This would not support the reverse‐sympatric model but seems substantially less likely (i.e., less parsimonious, rejected by ancestral reconstructions, and inconsistent with the pattern of mostly mixed populations surrounding LI). Similarly, another alternative explanation is that only one morph arrived first and then the color allele(s) for another morph arrived later, such that the history of color alleles was independent of the history of populations. This also seems unlikely, given that the populations of pure leadback morphs on LI clearly differ from other LI populations in many traits besides color (e.g., RADseq SNPs, microsatellites, mtDNA, morphology, macroclimate, microclimate). Furthermore, the idea that color alleles are independent of population history seems inconsistent with our phylogeny, showing pure redback and pure leadback clades on LI.

## 5 CONCLUSIONS

In summary, our results provide new phylogenomic evidence for an unusual scenario of potential incipient speciation, involving large‐scale spatial separation of formerly sympatric ecotypes into largely parapatric populations. Clues to the scenario in this system were apparent even without molecular data (Williams et al., 1968), given that these ecotypes are easily distinguished by color pattern. Similar examples may be hiding in other systems where the different phenotypes are not so apparent, but this remains to be seen. Our results may also illustrate how range expansion can involve distinctive, pre‐existing phenotypes within the species' range.

## AUTHOR CONTRIBUTIONS

**Emily Buckingham:** Data curation (equal); formal analysis (equal); methodology (equal); software (equal); writing – review and editing (equal). **Jeffrey W. Streicher:** Conceptualization (equal); data curation (equal); formal analysis (equal); investigation (equal); methodology (equal); project administration (equal); software (equal); supervision (equal); visualization (equal); writing – original draft (equal). **M. Caitlin Fisher‐Reid:** Conceptualization (equal); investigation (equal); methodology (equal); resources (equal); writing – review and editing (equal). **Tereza Jezkova:** Methodology (equal); writing – review and editing (equal). **John J. Wiens:** Conceptualization (equal); funding acquisition (equal); investigation (equal); supervision (equal); writing – original draft (equal).

## CONFLICT OF INTEREST

The authors declare no competing interests.

## Supporting information


Appendix S1

## Data Availability

Appendix S1, Figure S1, and Tables S1–S3 are available as a file called Supplementary Information. R code, data, and analyses settings are available on Dryad as Files S1–S3 (https://doi.org/10.5061/dryad.6t1g1jx30).
