# Supplementary material for: Population genomic analyses support sympatric origins of parapatric morphs in a salamander
Source: Ecol Evol. 2022 Nov 27;12(11):e9537. doi: 10.1002/ece3.9537 (PMC9702563; doi:10.1002/ece3.9537)
Supplement: Supplementary file 1 — Appendix S1 [file ECE3-12-e9537-s001.docx]

**Supplementary Information**

**Population genomic analyses support sympatric origins of parapatric morphs in a salamander**

Emily Buckingham^1,2*^, Jeffrey W. Streicher^1,3*^, M. Caitlin Fisher-Reid^4^, Tereza Jezkova^5^ and John J. Wiens^3^

^1^Department of Life Sciences, The Natural History Museum, London, UK

^2^Department of Life Sciences, Imperial College London (South Kensington), London, UK

^3^Department of Ecology and Evolutionary Biology, University of Arizona, Tucson, Arizona, USA

^4^Department of Biological Sciences, Bridgewater State University, Bridgewater, Massachusetts, USA

^5^Department of Biology, Miami University, Oxford, Ohio, USA

*These authors contributed equally

**This file contains:**

**Appendix S1.** Supplementary methods and results.

**Figure S1.** Results of SNAPP analysis conducted using only one SNP per RAD locus.

**Table S1.** Sampling of *Plethodon cinereus* used in the present study.

**Table S2.** Resulting statistics from missing data directionality index sensitivity analyses.

**Table S3.** Pairwise fixation indices (F_ST_ scores) among clades and populations of *Plethodon cinereus*.

**Files for Dryad (not in this file):**

**File S1.** Zipped directory containing R commands and datasets for generating figures and analyses.

**File S2.** Input file with data and analysis settings (XML format) for SNAPP analysis of *Plethodon cinereus* from Long Island and adjacent mainland populations using all SNPs.

**File S3.** Input file with data and analysis settings (XML format) for SNAPP analysis of *Plethodon cinereus* from Long Island and adjacent mainland populations using first-SNP only.

*Raw data from Illumunia Hi-Seq will be uploaded to the NCBI sequence read archive (SRA)

**Appendix S1.** **Supplementary methods and results.**

**(a) Potential batch effects**

Non-overlapping size selection between our primary dataset of 56 samples and the 13 additional samples, meant that they had few RAD loci in common and therefore could not be aligned for phylogenetic or range-expansion analysis (without obvious methodological artifacts in resulting analyses). However, we assumed that within-individual qualities (like observed heterozygosity) would be comparable between different size-selection protocols. The reason for this was two-fold. First, within-individual heterozygosity can be accurately estimated with hundreds of loci (Miller et al., 2014). Here, we used >500,000 sites per individual derived from 48,228 RAD loci. Second, these estimates do not require between-individual comparisons and therefore bypass any RADseq ‘batch effects’ in downstream analyses that would arise from inter-individual comparison (O’Leary et al., 2018; Lambert et al., 2019).

In contrast to estimating heterozygosity, using non-overlapping RAD libraries may introduce methodological artifacts when estimating numbers of private alleles. This is because the identification of private alleles is dependent upon a global database of allelic variation. Thus, more ‘private alleles’ may be identified in size-selection groups with fewer individuals sampled. To test for this effect in our dataset, we analysed all 90 samples using the populations program in STACKS 2.41 and then tested whether there were significantly different numbers of private alleles between the size-selection groups. Using non-parametric Kruskall-Wallis tests we found that there was a significant difference, with the smaller size-selection group having significantly more private alleles (H=14.23, *P*=0.0001). However, this result is difficult to interpret clearly as the smaller, supplemental size-selection group was comprised of ~41% mainland taxa, whereas the main text dataset only included ~27% mainland taxa.

The number of private alleles did not differ between size-selection groups for mainland individuals (H=1.37, *P*=0.2423), but did differ for LI individuals (H=16.71, *P*<0.0001). The supplemental size-selection group had more private alleles. We then removed the supplemental size-selection group and re-ran the analyses described above. We found that the reduced dataset supported significantly fewer private alleles on LI compared to the mainland (H=25.76, *P*<0.0001) and significantly more private alleles in pure redback populations on LI compared to their polymorphic and pure leadback counterparts (H=7.55, *P*=0.0230). Thus, while we detected evidence consistent with ‘batch effects’, the two findings we report for number of private alleles, were robust to these effects, specifically: (i) lower numbers of private alleles on LI (Fig. 3) and (ii) higher numbers of private alleles in LI pure redback populations (Fig. 3).

**(b) Reconstructing range expansion and missing data**

RADseq datasets typically have missing data, given both biological and methodological causes (Crotti et al., 2019). Prior to our study, it was not clear if missing data would impact the analyses conducted using rangeExpansion (Peter and Slatkin 2013). Therefore, we conducted sensitivity analyses to investigate the impact of various levels of missing data (Buckingham, 2019). For generating different levels of missing data, we used the approach of Crotti et al. (2019) and created three datasets. These datasets allowed the inclusion of SNPs with up to (i) 25%, (ii) 50%, and (iii) 75% missing individuals per SNP, respectively. We generated datasets for these three missing-data levels using the same STACKS pipeline described above. These datasets included 9,690 SNPs (only SNPs with up to 25% missing data included), 23,249 SNPs (50%), and 38,977 SNPs (75%). Thus, datasets with more SNPs had a higher percentage of missing data.

As with any empirical dataset, it was difficult to compare the performance of each dataset because the true range expansion history of *P. cinereus* on LI is unknown. Given that our primary concern was that missing data might obscure the signal of range expansion, we used comparisons with the most complete dataset (25% missing data) to understand how datasets with more missing data (50% and 75%) influenced three statistics generated by rangeExpansion (Peter & Slatkin, 2013). These statistics were: (i) the strength of the inferred founder effect (*q*), (ii) the inferred geographical coordinates of the origin of range expansion, and (iii) the coefficient of determination (*r*^2^) for the relationship between pairwise ψ scores and pairwise geographic distances. All three datasets had comparable founder effect signals (*q*=<0.001). The estimated location of range expansion origin was identical for the 50% and 75% missing datasets and was located 14.97 km east of the coordinates estimated from the 25% missing dataset. Both locations were in central western LI (Table S2). We found that *r*^2^ values increased with increasing number of SNPs and increasing missing data levels. This pattern was found in tests with all the samples included (25% missing: *r*^2^=0.048; 50% missing: *r*^2^=0.061; 75% missing: *r*^2^=0.085). However, none of these correlations were significant (Table S2).

We observed similar patterns after removing four populations that were >50 km from the centre of LI. These patterns included (i) more easterly expansion origins in the 50% and 75% missing datasets than in the 25% missing dataset (by 18.23 km) and (ii) an overall increase in *r*^2^ values (25% missing*: r*^2^=0.121; 50% missing; *r*^2^=0.150; 75% missing; *r*^2^=0.186). However, without the geographical outliers, *r*^2^ values were higher and significant (*P*<0.018) for all datasets (Table S2). Additionally, the strength of the inferred founder effect decreased slightly with increasing amounts of missing data (25% missing, *q*=0.003 versus 50% and 75% missing, *q*=0.002).

In summary, the datasets with more SNPs but higher levels of missing data produced higher *r*^2^ values (indicative of a stronger relationship between pairwise ψ scores and pairwise geographic distances), but sometimes had weaker signal of founder effects, compared to more complete datasets. Despite these differences, all analyses were largely congruent in where they predicted the origin of expansion to occur (Table S2), and all these origin estimates were further west than the geographical midpoint of our sampling (40.917110 N, 72.7711665 W; estimated from the midpoint of our eastern-most [Mont] and western-most [OldW] localities). These results are consistent with our other analyses (phylogeny, private alleles; see Results) which supported expansion from western LI. Thus, range expansion reconstruction [5] seems to be robust to different levels of missing data and spatial sampling (based on the removal of four extralimital populations). We used the most complete dataset (allowing up to 25% missing data per SNP) with four populations removed in the analyses discussed in the main text (Fig. 4). However, the significant results described are also recovered when using results from the other thresholds of missing data.

**References**

Buckingham, E. (2019). Range expansion of the Redbacked salamander (*Plethodon cinereus*) in Long Island, New York. Biosystematics MRes thesis. Imperial College London.

Crotti, M., Barratt, C.D., Loader, S.P., Gower, D.J. & Streicher, J.W. (2019). Causes and analytical impacts of missing data in RADseq phylogenetics: insights from an African frog (*Afrixalus*). *Zoologica Scripta* *48,* 157–167.

Lambert, S.M., Streicher, J.W., Fisher-Reid, M.C., Méndez de la Cruz, F.R., Martínez-Méndez, N., García-Vázquez, U.O., Nieto-Montes de Oca, A. & Wiens, J.J. (2019). Inferring introgression using RADseq and DFOIL: power and pitfalls revealed in a case study of spiny lizards (*Sceloporus*). *Molecular Ecology Resources* *19,* 818–837.

Miller, J.M., Malenfant, R.M., David, P., Davis, C.S., Poissant, J., Hogg, J.T., Festa-Bianchet, M., Coltman, D.W. (2014). Estimating genome-wide heterozygosity: effects of demographic history and marker type. *Heredity 112,* 240–247.

O’Leary, S.J., Puritz, J.B., Willis, S.C., Hollenbeck, C.M. & Portnoy, D.S. (2018). These aren’t the loci you’re looking for: Principles of effective SNP filtering for molecular ecologists. *Molecular Ecology 27,* 3193–3206.

Peter, B.M. & Slatkin, M. (2013). Detecting range expansions from genetic data. *Evolution 67,* 3274–3289.

**Figure S1.** Population-level phylogeny of *Plethodon cinereus* from Long Island (LI) and adjacent regions, The tree is inferred using the method SNAPP from 48,228 SNPs identified from ddRADseq data. (A) Densitree overview with branch lengths equivalent to expected mutations and (B) majority-rule consensus tree with posterior probabilities (with arbitrary branch lengths). Red dots indicate pure redback populations, black indicates pure leadback populations and teal indicates populations with both morphs. Labelled clades (C1–C4) are discussed in the text. Locality data are in Table S1.


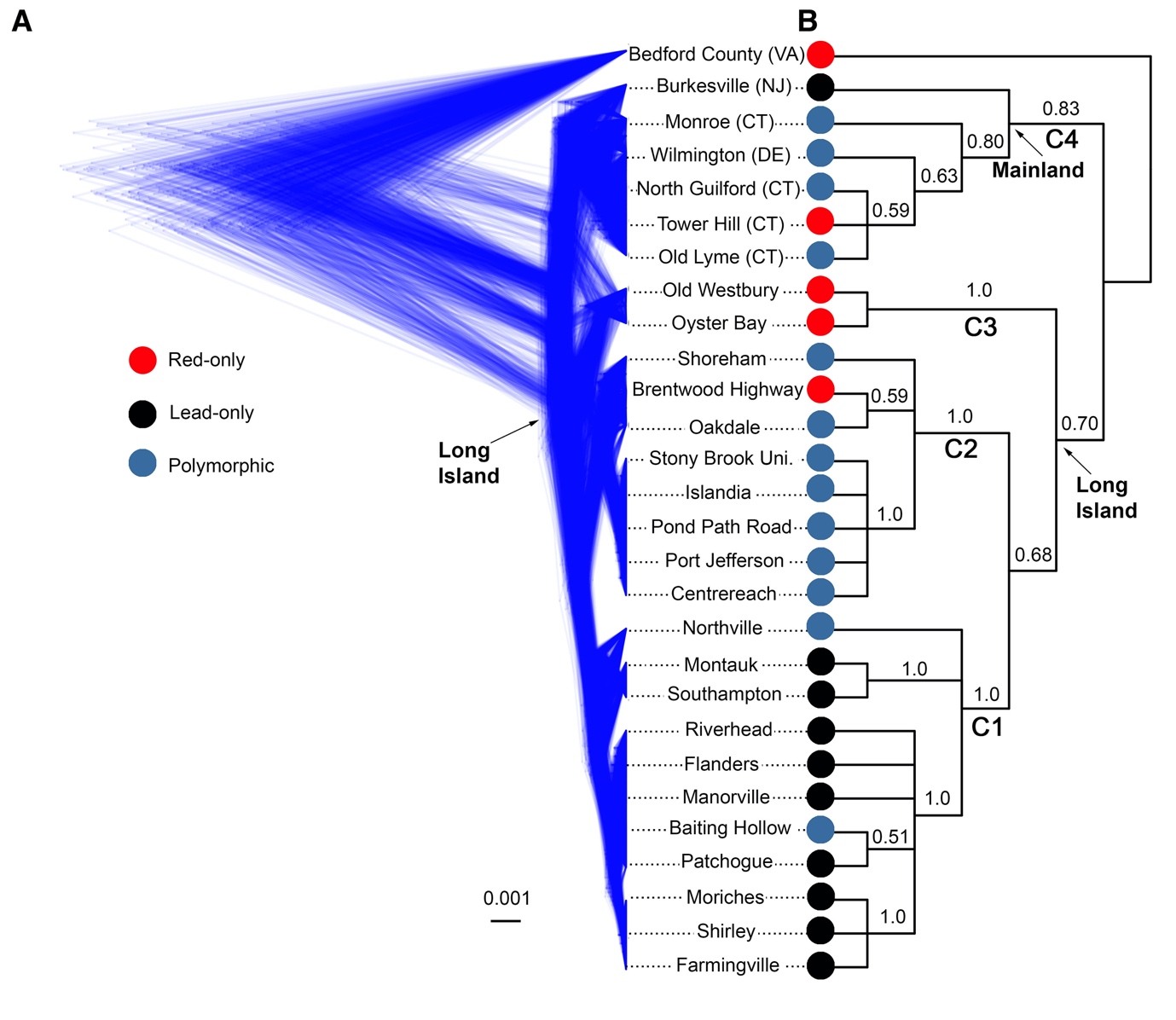


**Table S1.** Sampling of *Plethodon cinereus* used in the present study. Population gives the general location of the site (e.g. nearest town), whereas the latitude and longitude give the specific location. The number of individuals sampled per site is *n*. Sample IDs are from the field series of J.J. Wiens. Morph indicates whether the individual belongs to the redback morph (Red) or the leadback morph (Lead). Number of RAD sites is the total number of nucleotides analysed for each individual. Number of private alleles indicates number of unique single nucleotide polymorphisms observed in each individual. Observed heterozygosity is the proportion of sites that are heterozygous for each individual. An asterisk indicates samples used in the phylogenetic analysis and a psi (ψ) indicates samples used in the range-expansion analysis. A double asterisk indicates samples that were included in the phylogenetic/range analysis but not in the estimates of observed heterozygosity and the number of private alleles. Abbreviations (used in the input data files) are found below their respective localities.

| Population | Latitude | Longitude | *n* | Sample IDs | Morph | Number of RAD sites | Number of private alleles | Observed heterozygosity |
| --- | --- | --- | --- | --- | --- | --- | --- | --- |
| **Long Island** |  |  |  |  |  |  |  |  |
| Manorville  Mano* ^ψ^ | 40.874717 | -72.804117 | 2 | JJW 1081  JJW 1085 | Lead  Lead | 1489105  1484220 | 13  6 | 0.0003  0.0003 |
| Calverton  Calv | 40.899933 | -72.741333 | 2 | JJW 1094  JJW 1095 | Lead  Lead | 1258492  1723434 | 11  86 | 0.0002  0.0002 |
| Patchogue  Patc* ^ψ^ | 40.831883 | -72.935950 | 2 | JJW 1113  JJW 1123 | Lead  Lead | 1341822  1444714 | 9  4 | 0.0006  0.0004 |
| Southampton  Sout* | 40.915416 | -72.411222 | 2 | JJW 1148  JJW 1150 | Lead  Lead | 1367497  1356015 | 12  9 | 0.0004  0.0004 |
| Flanders  Flan* ^ψ^ | 40.900017 | -72.595050 | 2 | JJW 1165  JJW 1172** | Lead  Lead | 1515849  68170 | 9  2 | 0.0004  0.0003 |
| Montauk  Mont* | 41.030617 | -71.977500 | 2 | JJW 1181  JJW 1184 | Lead  Lead | 1451979  1474522 | 7  7 | 0.0003  0.0004 |
| Easthampton  EasH | 40.954617 | -72.228850 | 1 | JJW 1189 | Lead | 1180779 | 18 | 0.0001 |
| Coram  Cora | 40.849550 | -72.975050 | 1 | JJW 1876 | Lead | 1251785 | 13 | 0.0001 |
| Farmingville  Farm* ^ψ^ | 40.832750 | -72.994867 | 2 | JJW 1891  JJW 1892 | Lead  Lead | 1504206  1518193 | 11  1 | 0.0003  0.0003 |
| Shirley  Shir*^ψ^ | 40.794300 | -72.908917 | 2 | JJW 1899  JJW 1900 | Lead  Lead | 1292453  1409629 | 9  7 | 0.0002  0.0003 |
| Moriches  Mori* ^ψ^ | 40.821983 | -72.813433 | 2 | JJW 1916  JJW 1917 | Lead  Lead | 1513754  1492718 | 4  7 | 0.0003  0.0003 |
| Eastport  EasP | 40.844117 | -72.721000 | 1 | JJW 1934 | Lead | 1527032 | 45 | 0.0002 |
| Riverhead  RivA* ^ψ^ | 40.875850 | -72.638283 | 2 | JJW 1946  JJW 1947** | Lead  Lead | 1366340  131610 | 5  0 | 0.0004  0.0002 |
| Centrereach  CenR* ^ψ^ | 40.83225 | -73.079583 | 2 | JJW 1108  JJW 1109 | Red  Lead | 1483904  1502156 | 7  11 | 0.0003  0.0003 |
| Port Jefferson  Port* ^ψ^ | 40.931450 | -73.029350 | 2 | JJW 1258  JJW 1260 | Red  Lead | 1252012  1468828 | 10  7 | 0.0002  0.0003 |
| Oakdale  Oakd* ^ψ^ | 40.74935 | -73.127217 | 2 | JJW 1323  JJW 1326 | Red  Lead | 1478549  1514123 | 8  10 | 0.0004  0.0005 |
| Northville  Nort* ^ψ^ | 40.968000 | -72.616150 | 2 | JJW 2052  JJW 2054 | Lead  Red | 1509998  1480889 | 10  9 | 0.0004  0.0004 |
| Pond Path Road  Pond ^ψ^ | 40.91165 | -73.104933 | 2 | JJW 1306  JJW 1308 | Red  Lead | 1454047  1484513 | 6  6 | 0.0003  0.0003 |
| Stony Brook University  SBUA* | 40.912333 | -73.118167 | 1 | JJW 1383  JJW 1386** | Red  Lead | 1044456  187575 | 11  0 | 0.0002  0.0001 |
| Shoreham  Shor* ^ψ^ | 40.943067 | -72.856033 | 2 | JJW 1400  JJW 1401 | Lead  Red | 1404376  1313671 | 8  5 | 0.0003  0.0002 |
| Cutchogue  Cutc | 41.01447 | -72.51235 | 1 | JJW 1416 | Red | 1335051 | 21 | 0.0002 |
| Baiting Hollow  Bait* ^ψ^ | 40.95845 | -72.768767 | 2 | JJW 1444  JJW 1453 | Lead  Red | 1411526  1369467 | 4  1 | 0.0004  0.0005 |
| Islandia  Isla* | 40.813100 | -73.158683 | 2 | JJW 1497  JJW 1500 | Red  Lead | 1484777  1348066 | 7  29 | 0.0003  0.0001 |
| Selden  Seld ^ψ^ | 40.914017 | -73.032050 | 1 | JJW 1861 | Red | 1243316 | 38 | 0.0004 |
| Woodbury  Wood ^ψ^ | 40.830417 | -73.458883 | 1 | JJW 1331 | Red | 1489958 | 94 | 0.0002 |
| Oyster Bay  Oyst* ^ψ^ | 40.859483 | -73.516900 | 2 | JJW 1348  JJW1349 | Red  Red | 1322062  900225 | 15  7 | 0.0004  0.0002 |
| West Hills  WeHi | 40.800983 | -73.419800 | 1 | JJW 1359 | Red | 1271454 | 25 | 0.0002 |
| Old Westbury  OldW* ^ψ^ | 40.798050 | -73.564833 | 2 | JJW 2013  JJW 2015 | Red  Red | 883902  1054145 | 33  238 | 0.0002  0.0002 |
| Smithtown  SmiA | 40.8606 | -73.21593 | 1 | JJW 2024 | Red | 908069 | 37 | 0.0002 |
| Brentwood Highway  High** ^ψ^ | 40.791167 | -73.301117 | 2 | JJW 1365**  JJW 1366** | Red  Red | 33771  N/A | 1  N/A | 0.0002  N/A |
|  |  |  |  |  |  |  |  |  |
| **Mainland** |  |  |  |  |  |  |  |  |
| Wilmington, Delaware  DEWi* | 39.8079 | -75.5391 | 2 | JJW 1959  JJW 1962 | Red  Lead | 962133  1010727 | 44  50 | 0.0007  0.0007 |
| Burkesville, New Jersey  NJBu* | 40.14953 | -74.4397 | 2 | JJW 1486  JJW 1487 | Lead  Lead | 1271192  1107467 | 21  29 | 0.0004  0.0004 |
| Dunnfield Creek, New Jersey  NJDu | 40.972133 | -75.1257 | 1 | JJW 1835 | Red | 1134441 | 41 | 0.0007 |
| New Jersey  NJLa | 40.417317 | -74.306700 | 2 | JJW 1511  JJW 1512 | Red  Lead | 1175540  939665 | 12  28 | 0.0004  0.0002 |
| Princeton, New Jersey  NJPr | 40.915416 | -72.411222 | 2 | JJW 1541  JJW 1542 | Lead  Red | 1675479  1040554 | 149  36 | 0.0003  0.0003 |
| Monroe, Connecticut  CTMo* | 41.3361 | -73.22636 | 2 | JJW 1551  JJW 1564 | Red  Lead | 1114641  1233285 | 31  34 | 0.0006  0.0006 |
| Old Lyme, Connecticut  CTOL* | 41.33468 | -72.31583 | 1 | JJW 1573  JJW 1567** | Lead  Red | 1189089  39525 | 22  1 | 0.0007  0.0004 |
| North Guilford, Connecticut  CTNG* | 41.361617 | -72.691833 | 2 | JJW 1579  JJW 1595 | Red  Lead | 1270712  1383987 | 45  33 | 0.0006  0.0007 |
| Tower Hill, Connecticut  CTTH* | 41.359850 | -72.512167 | 2 | JJW 1604  JJW 1606 | Red  Red | 1262647  1764094 | 26  418 | 0.0007  0.0004 |
| Sleepy Hollow, New York  NYSH | 41.0953 | -73.83763 | 1 | JJW 1814 | Red | 921144 | 22 | 0.0002 |
| Orangeburg, New York  NYOr | 41.0626 | -73.94008 | 1 | JJW 1825 | Red | 1589129 | 2631 | 0.0003 |
| Westmoreland, Pennsylvania  PAWe | 40.3626 | -79.05545 | 1 | JJW 1838 | Red | 918573 | 328 | 0.0003 |
| Virginia  VA01 | 36.68875 | -81.651533 | 1 | JJW 1645 | Red | 924219 | 469 | 0.0008 |
| Bedford County, Virginia  VABC* | 37.458867 | -79.629 | 2 | JJW 1793  JJW 1794 | Red Red | 930265  937188 | 346  290 | 0.0010  0.0010 |
|  |  |  |  |  |  |  |  |  |

**Table S2**. Results of range explanation analyses, testing the impacts of missing data. Analyses were run under two conditions for populations of *Plethodon cinereus* from Long Island: (i) using all eligible samples of *P. cinereus* (‘Maximum sampling’) and (ii) using only samples within the dataset located >50km from the centre of Long Island (‘Outliers removed’). Missing data threshold is the maximum number of missing SNPs per site allowed in each dataset. Range expansion origin is the estimated location of where ancestral range expansion began. *q* indicates the strength of the founder effect (see main text). *r_1_*, *r_10_*, and *r_100_* show the decrease in diversity over 1km, 10km, and 100km, respectively. *r^2^* and *P* show the coefficient of determination and *P*-value for the estimated origin coordinates, respectively. Bolded *P*-values indicate statistical significance when 𝛼 = 0.05.

|  | Missing data threshold | Range expansion origin | *q* | *r_1_* | *r_10_* | *r_100_* | *r^2^* | *P* |
| --- | --- | --- | --- | --- | --- | --- | --- | --- |
|  |  |  |  |  |  |  |  |  |
| Maximum sampling  (23 populations) | 25% | 40.80856 N, 73.21209 W | <0.001 | 0.998 | 0.981 | 0.834 | 0.048 | 8.488 |
|  | 50% | 40.82337 N, 73.03572 W | <0.001 | 0.998 | 0.983 | 0.852 | 0.061 | 1.764 |
|  | 75% | 40.82337 N, 73.03572 W | <0.001 | 0.998 | 0.983 | 0.854 | 0.085 | 0.107 |
|  |  |  |  |  |  |  |  |  |
| Outliers removed (19 populations) | 25% | 40.80689 N, 73.24157 W | 0.003 | 0.995 | 0.952 | 0.665 | 0.121 | **0.018** |
|  | 50% | 40.79538 N, 73.02606 W | 0.002 | 0.995 | 0.956 | 0.683 | 0.150 | **0.001** |
|  | 75% | 40.80689 N, 73.02606 W | 0.002 | 0.996 | 0.959 | 0.700 | 0.186 | **<0.001** |
|  |  |  |  |  |  |  |  |  |

**Table S3.** Pairwise fixation indices (F_ST_ scores) among populations. Comparisons are shown (i) among and within four major clades and (ii) between mainland populations and between parapatric popluations on Long Island. Clade names C1–C3 refer to designations in Fig. 2 of the main text.

| Group | Number of comparisons | Average F_ST_ (S.D.) | Kruskal-Wallis (0.05) |
| --- | --- | --- | --- |
|  |  |  |  |
| Within clades | 110 | 0.242 (0.043) | H = 171.9,  *P* <0.00001*** |
| Between clades | 268 | 0.342 (0.041) |  |
|  |  |  |  |
| Between mainland populations (C4) | 15 | 0.263 (0.037) | H = 26.2,  *P* <0.00001*** |
| Between parapatric LI groups (C3-redback vs. C2-polymorphic and C1-mostly leadback vs C2-polymorphic) | 90 | 0.340 (0.039) |  |
|  |  |  |  |
